# Vibrational spectroscopy: a promising approach to discriminate neurodegenerative disorders

**DOI:** 10.1186/s13024-018-0252-x

**Published:** 2018-05-02

**Authors:** Maria Paraskevaidi, Pierre L. Martin-Hirsch, Francis L. Martin

**Affiliations:** 1 School of Pharmacy and Biomedical Sciences, University of Central Lancashire, Preston, PR1 2HE UK; 2 Lancashire Teaching Hospitals NHS Foundation, Sharoe Green Unit, Fullwood, Preston, PR2 9HT UK

**Keywords:** Infrared spectroscopy, Raman spectroscopy, Neurodegenerative disease, Diagnosis

## Abstract

Neurodegenerative diseases are a growing burden in modern society, thus crucially calling for the development of accurate diagnostic strategies. These diseases are currently incurable, a fact which has been attributed to their late diagnosis, after brain damage has already become widespread. An earlier and improved diagnosis is necessary for the enrolment of patients into clinical trials and can pave the way for the development of therapeutic tactics. Novel analytical techniques, such as mass spectrometry and vibrational spectroscopy, have been able to successfully detect and characterise neurodegenerative disorders. It is critical to globally support and make use of innovative basic research and techniques, which could ultimately lead to the creation of a cost-effective diagnostic test. Minimally invasive samples, such as biological fluids, have also been shown to reveal information for these diseases; utilising them could simplify sample collection/analysis and be more preferable to patients.

## Background

In recent years, research has made great strides in deciphering the underlying mechanisms of neurodegenerative diseases. It is now widely accepted that brain changes associated with the disease commence years prior to symptomatology, when preventative / therapeutic strategies are more likely to successfully intervene and decelerate, if not cease, disease progression. The fact that neurodegeneration is a slowly progressive disease can allow enough time for a series of screening tests and potentially a timely diagnosis to be made. Nonetheless, currently a definitive diagnosis can only be provided post-mortem and a robust means for early diagnosis remains yet to be established.

## Main text

As the percentage of the population over 65 years is growing, so is the burden of neurodegenerative disorders. An estimated 50 million people worldwide live with dementia, with this number getting doubled every 20 years, expected to reach 75 million in 2030 and 131.5 million in 2050 [1]. The major and enduring healthcare costs for neurodegenerative disease sufferers and their families are prohibitive in many countries and their respective health systems. For instance, the worldwide economic burden of dementia patients alone has been estimated to rise above a US$ trillion. It is evident that an accurate and cost-effective screening approach could permit the development of early preventative strategies that could impact on the disease progression.

**Fig. 1:**
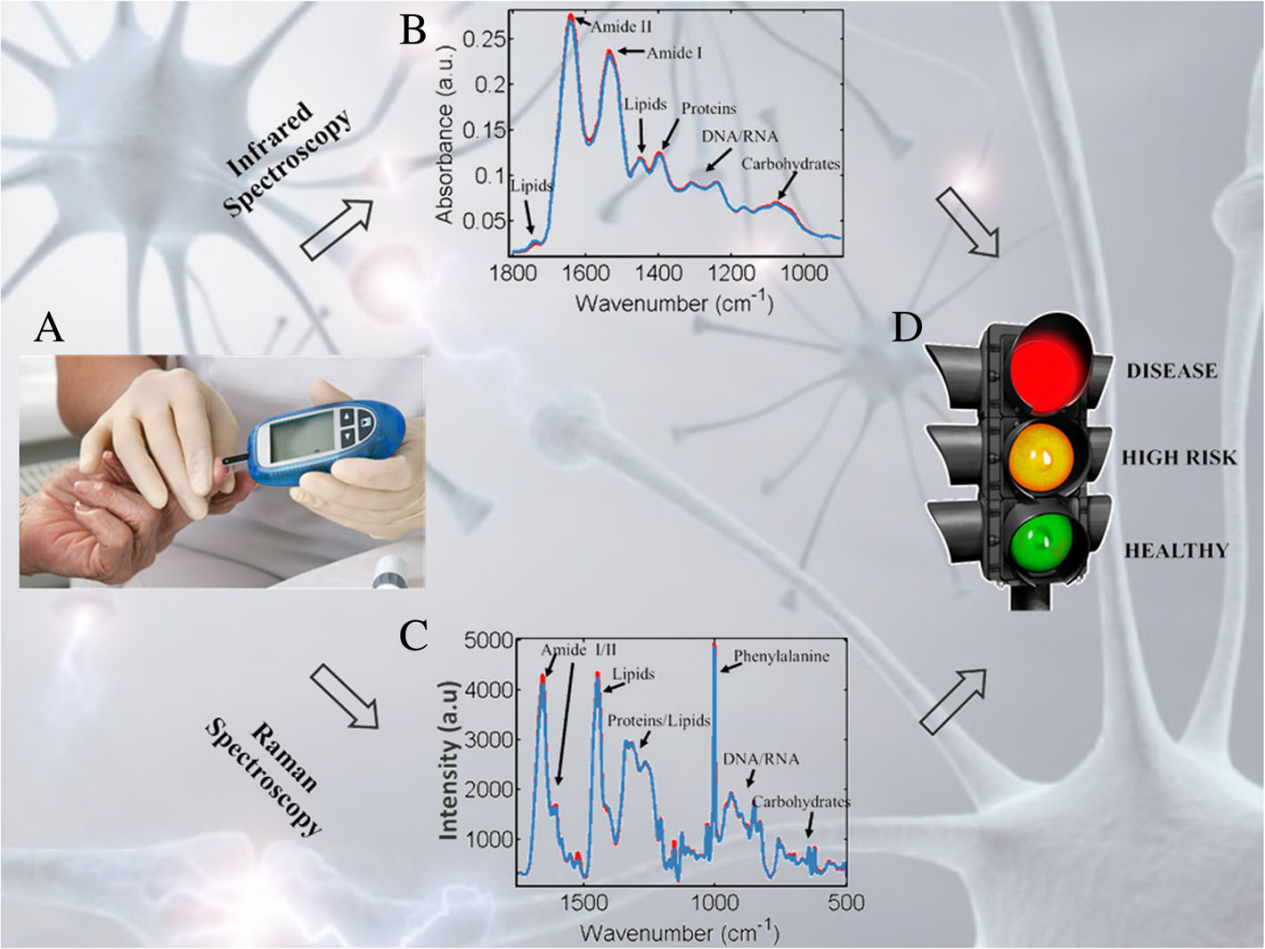
The potential of biospectroscopy in a clinical diagnostic pathway. **a** A handheld device is used to analyse the sample (e.g., blood or CSF) causing vibrations to its molecules. **b** Infrared and (**c**) Raman spectroscopies generate characteristic spectra which allow the identification of biomarkers indicative of disease. **d** Spectral analysis then follows to classify the patients into different categories using a traffic-light system: red suggesting disease, amber proposing high risk and further investigation and green indicate absence of disease

Two decades ago, the first evidence of the structure of β-amyloid plaques was revealed by synchrotron infrared (IR) microspectroscopy, after in situ analysis of the brain of an Alzheimer’s disease patient [2]. Today, technological improvements and new techniques, such as mass spectrometry or vibrational and nuclear magnetic resonance spectroscopies, have emerged as promising detection tools in the field of neurodegeneration [3–6]. Vibrational spectroscopy, including IR and Raman, is by far the simplest method, requiring less sophisticated instrumentation and consumables, thus automatically decreasing the expenses and rendering a potential test easily translatable (Fig. 1). So far, numerous studies have demonstrated the efficacy of vibrational spectroscopy in identifying individuals with various degenerative conditions, such as dementia or Parkinson’s disease, with high levels of diagnostic accuracy [7, 8].

Previous research has focused on the development of panels of biomarkers to improve the diagnostic accuracy for neurodegenerative diseases. However, the majority of the employed techniques require either expensive or invasive and laborious methods, including imaging and cerebrospinal fluid testing. In contrast, vibrational spectroscopy has the ability to investigate and identify numerous biological molecules simultaneously, therefore providing a multiple marker test for an underlying pathology while being rapid and inexpensive. Using developed machine-learning algorithms, the system is “trained” to recognize unique spectral markers that indicate disease; thus, when unknown samples are introduced, it is possible to accurately assess them in a traffic-light manner, with amber and red light suggesting further investigation and disease, respectively, while a green light would indicate absence of disease. Accordingly, high-risk population, as well as people with mild cognitive impairment or a traumatic brain injury could be monitored by serial sampling, which can be easily achieved by minimally-invasive blood tests. A detection of brain changes early in the course of disease would allow individuals to enroll in potential clinical trials, therefore promoting drug development and benefiting patients. An important advantage of this traffic-light system is that there is no need for prior expertise in spectroscopy and spectral analysis, with a nurse or a clinical doctor being able to immediately interpret the result. A representative example is the use of Raman spectroscopy during brain cancer surgery, which can provide invaluable information to surgeons by defining surgical margins in patients with different grades of brain tumour severity [9].

## Conclusions

Spectroscopy of biological fluids, such as blood and cerebrospinal fluid, is anticipated to facilitate the development of novel, cost-effective diagnostic strategies and promote rapid primary care. Recently developed hand-held portable devices also facilitate point-of-care diagnostics which could significantly expedite clinical implementation. Use of such technologies could enhance the accuracy of neurological diagnosis and also categorize disease types. However, similar to any innovation pending translation in a clinical context, spectroscopy requires a series of validation steps, including large-scale studies and coordinated efforts across different, multidisciplinary research groups. Both scientific and clinical communities need to embrace non-traditional methodologies and redouble their efforts towards the repetition and validation of the latter in order to render them translatable in a clinical context. A vital step for success would be to share knowledge and research results in wide-ranging and free-access electronic platforms from where patients, researchers and doctors alike would be able to keep track with the latest advancements in the area of neurodegeneration.

## Abbreviations

IR: infrared
